# Immunoinformatics Features Linked to *Leishmania* Vaccine Development: Data Integration of Experimental and In Silico Studies

**DOI:** 10.3390/ijms18020371

**Published:** 2017-02-10

**Authors:** Rory C. F. Brito, Frederico G. Guimarães, João P. L. Velloso, Rodrigo Corrêa-Oliveira, Jeronimo C. Ruiz, Alexandre B. Reis, Daniela M. Resende

**Affiliations:** 1 Laboratório de Pesquisas Clínicas, Programa de Pós-graduação em Ciências Farmacêuticas/CiPharma, Escola de Farmácia, Campus Morro do Cruzeiro, Universidade Federal de Ouro Preto, Bauxita, 35.400-000 Ouro Preto, Minas Gerais, Brazil; rorybrito@gmail.com; 2 Laboratório de Imunopatologia, Núcleo de Pesquisas em Ciências Biológicas, Campus Morro do Cruzeiro, Universidade Federal de Ouro Preto, Bauxita, 35.400-000 Ouro Preto, Minas Gerais, Brazil; 3 Grupo Informática de Biossistemas e Genômica, Programa de Pós-graduação em Ciências da Saúde, Centro de Pesquisas René Rachou, Fiocruz Minas, Av. Augusto de Lima, 1715, Barro Preto, 30.190-002 Belo Horizonte, Minas Gerais, Brazil; frederico.guimaraes@cpqrr.fiocruz.br (F.G.G.); jpvlinhares@gmail.com (J.P.L.V.); jeronimo@cpqrr.fiocruz.br (J.C.R.); dmresende@cpqrr.fiocruz.br (D.M.R.); 4 Grupo Imunologia Celular e Molecular, Programa de Pós-graduação em Ciências da Saúde, Centro de Pesquisas René Rachou, Fiocruz Minas, Av. Augusto de Lima, 1715, Barro Preto, 30.190-002 Belo Horizonte, Minas Gerais, Brazil; correa@cpqrr.fiocruz.br; 5 Instituto Nacional de Ciência e Tecnologia em Doenças Tropicais (INCT-DT), Campus Morro do Cruzeiro, Universidade Federal de Ouro Preto, Bauxita, 35.400-000 Ouro Preto, Minas Gerais, Brazil; 6 Programa de Pós-graduação em Biologia Computacional e Sistemas, Instituto Oswaldo Cruz, Fiocruz, Av. Brasil, 4.365, Pavilhão Arthur Neiva, Manguinhos, 21.040-360 Rio de Janeiro, Rio de Janeiro, Brazil

**Keywords:** immunoinformatics, epitope prediction, pathways, protein–protein interaction networks, reverse vaccinology, leishmaniasis

## Abstract

Leishmaniasis is a wide-spectrum disease caused by parasites from *Leishmania* genus. There is no human vaccine available and it is considered by many studies as apotential effective tool for disease control. To discover novel antigens, computational programs have been used in reverse vaccinology strategies. In this work, we developed a validation antigen approach that integrates prediction of B and T cell epitopes, analysis of Protein-Protein Interaction (PPI) networks and metabolic pathways. We selected twenty candidate proteins from *Leishmania* tested in murine model, with experimental outcome published in the literature. The predictions for CD4^+^ and CD8^+^ T cell epitopes were correlated with protection in experimental outcomes. We also mapped immunogenic proteins on PPI networks in order to find Kyoto Encyclopedia of Genes and Genomes (KEGG) pathways associated with them. Our results suggest that non-protective antigens have lowest frequency of predicted T CD4^+^ and T CD8^+^ epitopes, compared with protective ones. T CD4^+^ and T CD8^+^ cells are more related to leishmaniasis protection in experimental outcomes than B cell predicted epitopes. Considering KEGG analysis, the proteins considered protective are connected to nodes with few pathways, including those associated with ribosome biosynthesis and purine metabolism.

## 1. Introduction

Leishmaniasis is a wide-spectrum disease caused by parasites from *Leishmania* genus. It is prevalent in Americas, Europe, Africa and Asia. Overall, human infection is caused by at least 20 species whose vectors are phlebotomine sandflies [1]. Although being considered by many studies one of the best possible alternatives for this disease control, there is no human vaccine available [2].

In the advent of reverse vaccinology, in the latest years, a great effort has been made by bioinformaticians in order to provide epitopes predictors programs. Currently, it is possible to scan entire genomes searching for immunogenic epitopes and then select promising proteins for vaccine development. The bottleneck in this workflow analysis is the validation of predictions for protozoan parasites. Many predictors are available for B cells, T CD4^+^ cells and T CD8^+^ cells epitopes and subcellular localization. They are valuable in a pre-screening evaluation for vaccine targets and searching for diagnostic markers.

The building of protein-protein interaction (PPI) networks may give some insights to understand the biological role of these targets, and so might be a valuable asset in vaccine development. These networks are constituted by nodes that correspond to proteins, connected by edges, representing the interactions between two connected proteins. With PPI networks, we can have an overview of protein relationships and notice those with high connections (also referred as “hubs”). Hub proteins tend to have essential role in the parasite metabolism and might be good candidates to vaccinal and drug target [3,4].

To support *Leishmania* vaccine research, we developed an approach that integrates prediction of B and T cell epitopes, analysis of PPI networks and metabolic pathways. With the aim of validating this methodology, we selected *Leishmania* proteins tested as vaccine candidates in murine model, with experimental outcome (EO) published in the literature. After predicting epitopes in the selected proteins using specific computational programs, we correlated the predictions for T CD4^+^ and T CD8^+^ cells with protection in EO. Finally, we mapped the immunogenic proteins on PPI networks in order to find Kyoto Encyclopedia of Genes and Genomes (KEGG) pathways associated with them.

## 2. Results

### 2.1. Leishmania Proteins Dataset Selection

Through the use of text mining technics from Pubmed website that included, but was not restricted to, categorization and entity extraction, we were able to identify and select 20 proteins from six different *Leishmania* species that were used in studies aiming the vaccine development against these parasites.

It is important to highlight that, for each one of those proteins, a specific MySQL ID was assigned to link GI accession number and TriTrypDB specific ID. Based on the results published, the EO was categorized into: (a) “no protection” (nine proteins); (b) “partial protection” (five proteins); and (c) “protection” (six proteins). The accession numbers of these proteins are depicted in Table 1.

### 2.2. Epitope and Subcellular Localization Predictions

With the purpose of selecting potential immunogenic epitopes in the selected experimental dataset, Structured Query Language (SQL) statements were used. The results obtained in terms of number of predicted binding Major Histocompatibility Complex (MHC) class I and II epitopes, B cell epitopes and subcellular locations are detailed in Table 2. Interestingly, the majority of the proteins within the “protection” group were predicted as extracellular and the proteins belonging to “no protection” group were predicted as located in nuclear and cytoplasmic compartments.

Specifically regarding the epitopes capacity to bind MHC class I, MHC class II and epitopes for B cell activation, considering a path from “no protection” to “protection” groups, a gradual increase of the number of predicted epitopes for T cells and B cells was observed.

### 2.3. Predicted Epitopes and Experimental Outcome Correlation

To evaluate the possible association between the number of predicted epitopes (NPE) for B and T cells, and the EO of selected proteins, the following consensus predictions were produced: (a) prediction for T CD8^+^ epitopes obtained from NetMHC and NetCTL; and (b) prediction for B cell epitopes obtained from AAP12, BCPred12 and BepiPred. The consensus predictions were obtained overlapping identical predictions made by different methodologies.

**Figure 1:**
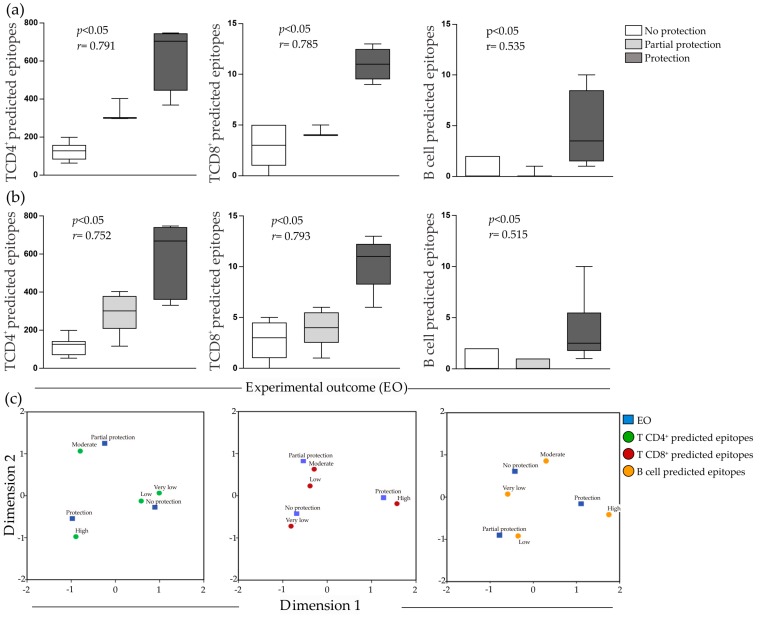
Correlation analysis: (**a**) Box plots of the relationships between T CD4^+^, T CD8^+^ and B cell epitopes and experimental outcome of candidate antigens taking into account cutaneous leishmaniasis (CL); (**b**) box plots of the relationships between T CD4^+^, T CD8^+^ and B cell epitopes and experimental outcome of candidate antigens concerning leishmaniasis diseases with no stratification; and (**c**) correspondence map showing the association between experimental outcome and T CD4^+^, T CD8^+^ and B cell predicted epitopes for leishmaniasis with no stratification.

To graphically depict the results, Box Plots and Correspondence Maps (CM) approaches were applied to visualize the potential associations determined through Spearman r and Chi-square distance, respectively. Firstly, the disease (leishmaniasis) was stratified into cutaneous leishmaniasis (CL) and visceral leishmaniasis (VL) and the EO of antigens from *Leishmania* that cause CL and VL were correlated with NPE (Figure 1a). Regarding VL analyses, significant correlation was observed only with EO and predicted epitopes for CD8^+^ T cells (*p* < 0.05) (data not shown). On the other hand, for CL analyses, it was observed significant correlation between EO and NPE for T CD4^+^, T CD8^+^ and B cells, as shown in Figure 1a. After that, analyses were performed concerning the disease without any stratification. As can be observed from Figure 1b (NPE and EO correlation for T CD4^+^, T CD8^+^ and B cells), a significant correlation exists between NPE specific to CD4^+^ and CD8^+^ T cells with *r* = 0.752/*p* < 0.05 and *r* = 0.793/*p* < 0.05. In addition, a weak association with B cell predicted epitopes (*r* = 0.515/*p* < 0.05) was observed. In other words, non-protective antigens have lowest frequency of predicted T CD4^+^ and T CD8^+^ epitopes, compared with protective ones.In regards to CM analysis (Figure 1c), considering the adopted variables (antigens EO versus NPE for T and B cells), the grouping outcome, which is related with data correlation, shows the same strong association above mentioned for leishmaniasis with no stratification.

As the last analysis layer used to validate data correlation, the Chi-square results confirmed the significant association between EO and the predicted epitopes for T CD4^+^ and T CD8^+^, *p* < 0.05 (Tables S1 and S2) and the weak one between EO and predicted epitopes for B cells (*p* < 0.05, see Table S3).

### 2.4. Number of Alleles (NA) and Experimental Outcome (EO) Correlation

**Figure 2:**
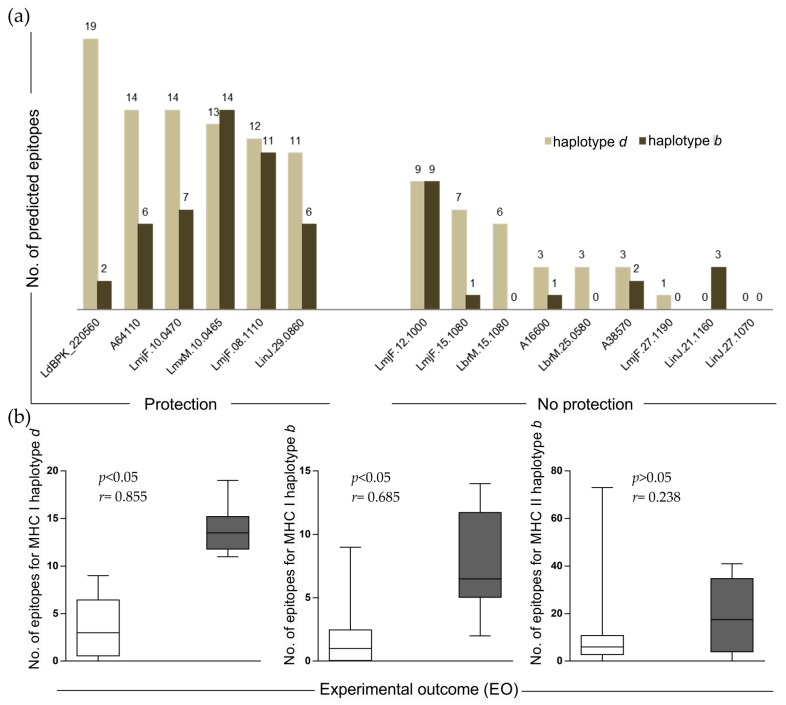
Evaluation of epitopes amount and relationship with experimental outcome: (**a**) bar graph showing number of epitopes for MHC class I and II (haplotype *d* and *b*) in the selected antigens classified in “protection” and “no protection” groups; and (**b**) box plot of the relationships between CD4^+^ and CD8^+^ T cell epitopes and experimental outcome of candidate antigens.

To hypothesize possible reasons linked with vaccine success or failure, an evaluation of allele-specific affinity (MHC I and II) was investigated. As illustrated in Figure 2a, the amounts of epitopes binding MHC haplotype *d* (BALB/c MHC alleles) and haplotype *b* binders (C57BL/6 MHC alleles) identified in the “protection” group were superior to those ones identified in the “no protection” group. In summary, our results indicate that epitopes for MHC class I and II haplotype *b* and *d* are more frequent in the success antigens tested for vaccine development. In addition, a detailed analysis in which MHC class I and II haplotypes were individually investigated revealed a strong association between NPE from MHC class I haplotype *d* and EO (*p* < 0.05 and *r* = 0.855) that is not observed for MHC class II haplotype *d* and *b*.

### 2.5. Mapping Immunogenic Proteins on Protein-Protein Interaction Networks (PPI Networks)

**Figure 3:**
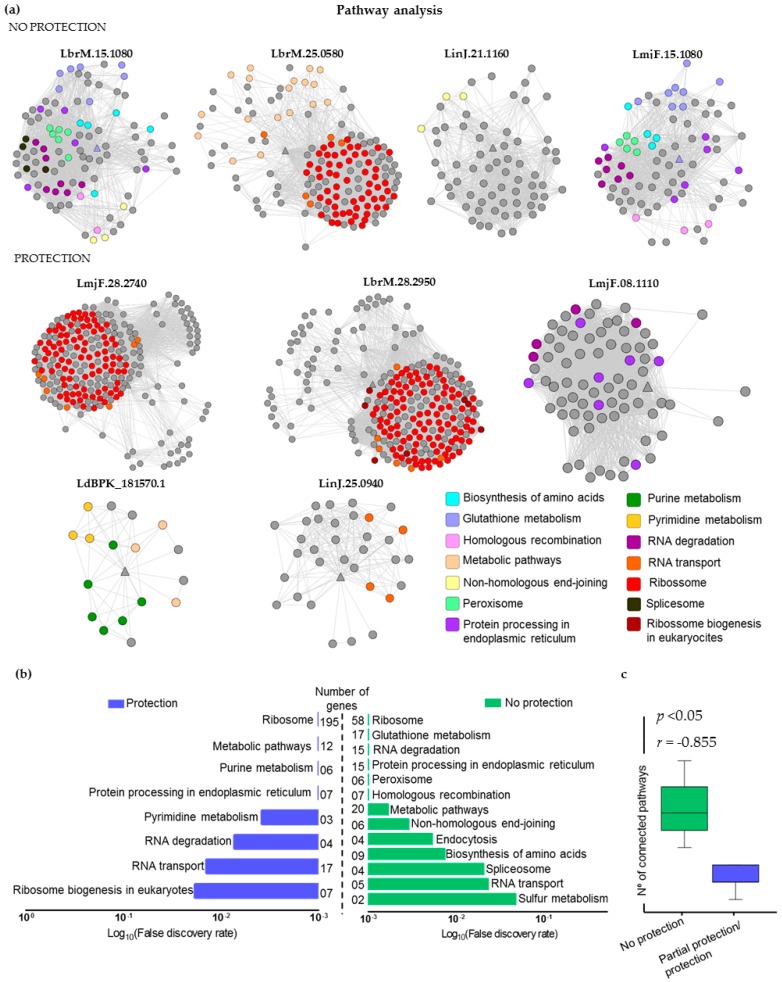
Immunogenic proteins mapped in Protein-Protein Interaction (PPI) networks: (**a**) PPI networks constructed starting with the target proteins (“no protection” and “partial protection/protection”) represented by triangles and specific pathways associated with each node (circles) using KEGG database. (**b**) Analysis of KEGG enriched pathways was performed by False Discovery Rate. For both “no protection” and “partial protection/protection”, the bar shows the fold-enrichment of the pathways. (**c**) Significant correlation between number of pathways connected with the target proteins of “no protection” and “partial protection/protection” groups (*p* = 0.007).

We chose Cytoscape to model PPI networks using data from STRING v.10. Figure 3a presents these networks, annotated according to their related KEGG pathways. Analyses of enriched pathways are shown in Figure 3b, according to their False Discovery Rate. Considering the “no protection” group, the most common pathways found were: ribosome (58 genes involved), glutathione metabolism (17 genes), RNA degradation (15 genes), protein processing in endoplasmic reticulum (15 genes), peroxisome (sixgenes) and homologous recombination (sevengenes). On the other hand, for “protection” group, we identified pathways as ribosome (195 genes involved), metabolic pathways (12 genes), purine metabolism (sixgenes) and protein processing in endoplasmic reticulum (seven genes). Interestingly, target proteins from “no protection” group are connected with nodes from many different pathways. In contrast, we observed that proteins of the “protection” group are connected to nodes of few pathways. Thus, there is a strong negative correlation (*r* = −8.55) between the number of connected pathways and EO of the selected proteins (Figure 3c).

## 3. Discussion

Nowadays, many computational methodologies have been described for epitope predictions of bacterial, fungal and others microorganisms. For protozoan (specifically *Leishmania* species), there are not strong and validated platforms to identify promising antigens for *Leishmania* vaccines [23]. Herein, we developed a sturdy and complete platform with potential of identifying candidates for vaccines against leishmaniasis. This platform integrates prediction of B and T cell epitopes, analysis of PPI networks and signaling pathways.

The first step was to select the input antigens to validate the platform. In this concern, we did an extensive search in the literature regarding antigens tested in murine model. It was difficult to categorize the 20 selected antigens for this work because there is a lack of consensus concerning the model, challenge inoculum, mechanisms of protective immunity (response induction, parasite burden reduction), so there is no standardization to appoint if a vaccine indicates protection or not [24]. We tried to choose proteins with no interference of adjuvant since it can entirely modify the antigen response [25]. 

Regarding the platform validation, we performed the epitope mapping using ad-hoc algorithms. Our results suggest that antigens with more predicted epitopes for T CD4^+^ and T CD8^+^ cells could be associated with protection in EO. In this regard, our results revealed that there are strong correlation and association between predicted epitopes and the EO. The inertia values show the powerful association between the variables. The CM dimension 1 represents the highest inertia allowing the interpretation of the results in the first dimension. T CD4^+^ and T CD8^+^ predicted epitopes versus EO show higher inertia values when compared to B cells predicted epitopes emphasizing that epitopes for CD4^+^ and CD8^+^ T cells are crucial for *Leishmania* vaccines success. We used all available human and mouse alleles to restrict the epitopes allowing enhance the assertive prediction. This analysis is useful to identify conserved epitopes that can bind various alleles of MHC appointing for rare and promising epitopes. The in silico analyses and in vivo validation of epitopes demonstrates that some algorithms may be important tools for the identification of epitopes, and consequently of immunogenic proteins. The algorithm NetCTL version 1.2 makes prediction of peptide–MHC class I binding, proteasomal C terminal cleavage, both using artificial neural networks, and TAP transport efficiency using weight matrix. The tree predictions are then integrated [26]. Another predictor also used for MHC class I binding peptides was NetMHC version 3.0. It predicts binding of peptides to different HLA alleles using artificial neural networks and weight matrices. For peptide–MHC class II binding prediction NetMHCII, version 1.0, was used. It predicts binding of peptides to 14 different HLA-DR alleles, including human and mouse, using position specific weight matrices [27]. To perform B-cell epitopes predictions, we used only methods that predict continuous epitopes. We used first BepiPred, version 1.0, that predicts linear B-cell epitopes using a combination of Hidden Markov model and a propensity scale method [28]. Then we used BCPREDS server comprising the AAP12 and BCPred12 predictors. The first one is based on the finding that B-cell epitopes favor particular amino acid pair, and it was trained using support vector machine classifier. The second uses subsequence kernel trained using support vector machine classifiers with 701 linear B-cell epitopes, extracted from Bcipep database, and 701 non-epitopes, randomly extracted from SwissProt sequences [29,30,31]. Finally, we used WoLF PSORT predictor, which is an amino acid sequence predictor of subcellular localization sites of proteins. It uses known sorting signal motifs and some correlative sequence features [32]. The integration of these predictors could reveal proteins that are secreted or presented in parasite membrane, capable of eliciting B and T cells responses.

Herrera-Najera et al. [33] performed a large-scale prediction of T cell epitopes in the whole genome of *L. major*, obtaining 26 potential epitopes through prediction consensus. Fourteen of them revealed to be immunogenic epitopes that were capable to stimulate T cells to produce IFN-γ. Other studies employing computational predictions in specific *Leishmania* proteins have shown that it is quite possible to use combined algorithms in epitopes searching that could be validated by in vivo experiments [34]. Duarte et al. [35] developed a combined epitope prediction platform in order to investigate T CD8^+^ epitopes in 63 *L. braziliensis* proteins, demonstrating a cytotoxic activity of some predicted epitopes in *Leishmania* infected mice. Recently, in silico methods for linear epitope predictions (NetMHC, NetCTL, and NetMHCII) were combined with molecular modeling to identify potential epitopes in the whole *L. braziliensis* proteome. Therefore, the pipeline was validated based on stimulation of human peripheral blood mononuclear cells (PBMCs) proliferation. The results obtained after the in vitro assays showed that six of ten selected epitopes could be classified as potentially immunogenic [36].

The role of B cell epitopes still hasroom for discussion since there is no consensus if immunoglobulins could be associated with resistance or susceptibility in leishmaniasis [37,38]. On the other hand, our results demonstrate a correlation between protection and specific B cell epitopes. In this context, we suggest that an effective vaccine should have epitopes capable of eliciting a strong T cell response and B cells too. In addition to the epitopes for B cells and MHC class I and II, another important feature is the subcellular localization of the antigen. It is known that extracellular *Leishmania* proteins are more immunogenic and considered better targets for vaccine development [39,40]. This fact is indeed corroborated by our findings that show the majority of the antigens from “protection group” are linked with extracellular compartmentalization. In silico approaches have limitations regarding the proteome annotation (e.g., the data of *L. amazonensis* used in this work) and the large number of linear epitopes. Nevertheless, our results of epitope prediction indicate a higher assertive and successful prediction, so it can be a useful approach for vaccine development against leishmaniasis.

To better understand the biological importance of vaccine candidates, we proposed the use of PPI networks enriched with KEGG pathways information. It is well known that some proteins are essential for specific biological processes of *Leishmania* spp. [41,42]. In this context, we proposed to analyze antigen pathways through modeled PPI and its relation with protection and no protection of vaccine candidates after challenging with infective *Leishmania* parasites. Our analyses showed that many of the selected antigens do not have any KEGG pathway associated to them, but, instead, are connected to proteins that are part of some pathway. Pathways associated with ribosome biosynthesis, purine metabolism and metabolic processes are present in “protection” group networks. Ribosome related proteins were considered relevant molecules during infection, since in some circumstances they can modulate cell activities and cytokine release. Many works associated these pathways to immune response [43]. Cordeiro-Da-Silva and collaborators in 2001 characterized a *Leishmania major* gene considered to be homologous to the mammalian ribosomal protein S3a. This ribosomal protein can be found in many other *Leishmania* species such as *L. infantum*, *L. amazonensis*, and *L. mexicana.* The article authors suggested that this protein could participate in the Th1/Th2 immune response balance during leishmaniasis [44]. Soto and collaborators in 1993 using sera from dogs affected by visceral leishmaniasis identified high antigenic *Leishmania* acidic ribosomal proteins, also called P-type proteins [45]. Another work by Soto and collaborators in 2000 showed that intraperitoneal administration in BALB/c mice of the acidic ribosomal protein LiP2a, without adjuvants, elicited a strong humoral response and was capable of stimulating production of IFN-γ in cultured splenocytes from LiP2a-immunized mice [46]. Our findings also match with results obtained in the secretome of *L. donovani*, where the majority of virulent proteins (secreted proteins) belong mainly to metabolic and biosynthesis processes [47]. To check the importance of some proteins related to metabolic process, *Leishmania* knocked-out for protein kinases and phosphatases possible involved in parasite metabolism regulation were generated. After this process, in many cases highly attenuated or completely avirulent parasites could be observed [48]. Naderer and collaborators generated a *Leishmania major* mutant lacking the regulatory subunit of the Ca^2+^/calmodulin-dependent serine/threonine-specific phosphatase. This modified *Leishmania* grew normally at 27 °C. However, this parasite lost viability when exposed to 34 °C [49]. Target of rapamycin (TOR) kinases are involved in some regulatory pathways related to cell growth and structure in eukaryotes. Silva and collaborators generated TOR3 knocked-out *Leishmania major* parasites. These knocked-out parasites exhibited slower growth than wild-type parasites and were unable to survive or replicate in macrophages in vitro. These parasites were not capable of inducing disease or establish infection in mice in vivo [50]. In addition, McConville and Naderer [48] have shown that metabolic pathways are important to *Leishmania* virulence, since down regulation of metabolic genes causes latency of many *Leishmania* species. One possible application of these attenuated or avirulent parasites could be in whole parasites vaccines. Carter et al. [51] have noticed that purine metabolism is vital for *Leishmania* survival. Surprisingly, our findings show that proteins associated to protection are connected to few pathways when compared to proteins that are not protective.

In summary, in this work, we proposed and validated a computational approach regarding epitope prediction, topological structure and pathway analyses to drive a rational vaccine design against leishmaniasis.

## 4. Materials and Methods

### 4.1. Selection of the Leishmania Antigens

*Leishmania* proteins tested in murine model, with EO published in the literature, were selected. Studies describing vaccine effectiveness after challenge with *Leishmania* spp. were preferentially chosen. Bearing in mind that there is no standardization of the protection concept and that there is variation of results in the literature, it was necessary to create three categories as already described. According to our categorization, “no protection” group includes antigens that promote no or slight reduction of parasite burden or lesions after *Leishmania* challenge. The “protection” group includes antigens that promoted significantly strong reduction of the parasite burden or lesions showing strong immune response to *Leishmania* antigens. Thus, the term partial protection was used to classify antigens that are between a potential protection and no protection at all. The “partial protection” group comprises proteins that could slightly reduce the parasite burden and/or lesions more than “no protection” group. In addition, these proteins can elicit some immune response which results in ineffective protection. It is important to highlight that the influence of adjuvants was not taken into account for the categorization, thus only the response of antigens tested alone was chosen to categorize the groups. Twenty candidates from Old and New World *Leishmania* species were categorized according with their experimental results in the following groups: (a) “no protection”; (b) “partial protection”; and (c) “protection”. The selected experimentally validated data included information from proteins of *L. amazonensis*, *L. braziliensis*, *L. major*, *L. mexicana*, *L. donovani* and *L. infantum* that were subsequently used in this study to corroborate the in silico bioinformatics predictions. The selected antigens are described in Table 1.

### 4.2. Leishmania Proteome Data

The predicted proteome sequences of dermatotropic and viscerotropic *Leishmania* species were obtained from TriTrypDB (Kinetoplastid Genomics Resource) and the sequence of *L. amazonensis* was downloaded from http://bioinfo08.ibi.unicamp.br/leishmania/ [52]. Detailed information about the predicted proteomes versions used in this work can be found in Table 3.

### 4.3. Epitope and Subcellular Localization Predictions

All proteomic data used in this work were screened in order to predict T CD4^+^ and T CD8^+^ epitopes, B cell epitopes and subcellular localization of proteins.

**Figure 4:**
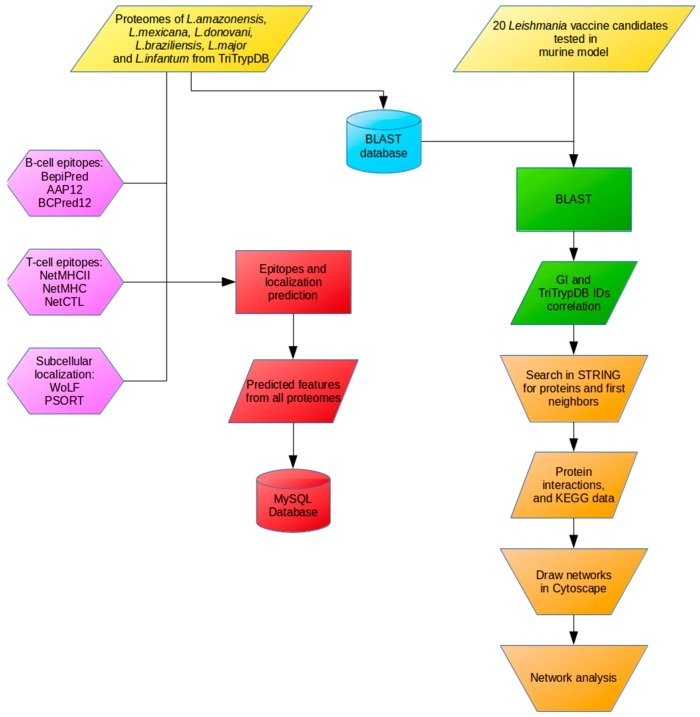
Workflow of analysis showing the steps followed along this work.

For T CD8^+^ epitope prediction (MHC class I binding epitopes), algorithms NetCTL [26,53,54] and NetMHC [55,56,57] were used. Regarding T CD4^+^ epitopes, NetMHCII [27,58] was used to predict MHC class II binding epitopes. For B cell epitopes, BepiPred [28] and BCPREDS (AAP12 and BCPred12 models) [29,30,31] were used to predict epitopes. Finally, the protein subcellular localization was predicted using WoLF PSORT [32,55,56,57]. The algorithms choice was made taking into account their viability for local stand-alone server installation, the number of citations found in literature, and the results previously published by Resende, Rezende, Oliveira, Batista, Correa-Oliveira, Reis and Ruiz [23] describing algorithms specificity, sensitivity and accuracy with parasite data obtained from UniProt (http://www.uniprot.org/) and IEDB (Immune Epitope Database and Analysis Resourse) (http://www.iedb.org/). The analytical workflow used in this study is presented in Figure 4.

To carry out the predictions, the algorithms were parameterized for eukaryotic genomes. For MHC class I binding epitopes, predictions for 12 human supertypes and seven mice alleles were performed. The following alleles were used: A1, A2, A3, A24, A26, B7, B8, B27, B39, B44, B58, B62, H2-Db, Dk-H2, H2-Dd, H2-Kb, H2-Kd, Kk-H2, and H2-Ld. Concerning MHC class II binding epitopes, we used 14 human alleles and three mice alleles bringing the total of different alleles to 17, as follows: HLA-DRB1*01:01, HLA-DRB1*03:01, HLA-DRB1*04:01, HLA-DRB1*04:04, HLA-DRB1*04:05, HLA-DRB1*07:01, HLA-DRB1*08:02, HLA-DRB1*09:01, HLA-DRB1*11:01, HLA-DRB1*13:02, HLA-DRB1*15:01, HLA-DRB3*01:01, HLA-DRB4*01:01, HLA-DRB5*01:01, H2-IAs, H2-IAd and H2-IAb. 

### 4.4. Development of Relational Database

**Figure 5:**
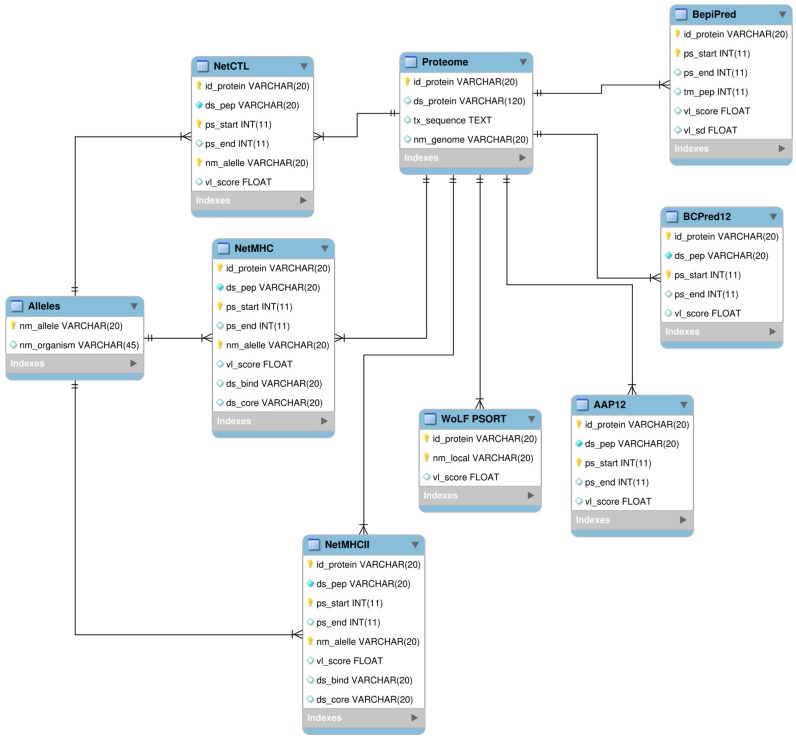
MySQL relational database scheme developed to integrate data from predictions.

Taking into account the great amount of data generated by the algorithms, we constructed a relational database using MySQL as Relational Database Management System (RDBMS) (http://www.mysql.com). The use of a database system in this work represents a crucial step that allows the integration of the results from all predictors and a way of getting a data receptacle or conceptual repository from which is possible to extract data correlation, helping in the identification of target proteins. The MySQL GUI Tools (http://dev.mysql.com/downloads/gui-tools/5.0.html) were used as a graphical user interface for our MySQL database. The relational model was built in MySQL Workbench (http://wb.mysql.com). To extract, parse and load data into database, specific Perl scripts were developed using DBI (the Perl interface to databases) and BioPerl modules. The relational schema is presented in Figure 5.

### 4.5. Mapping Immunogenic Proteins on Protein-Protein Interaction Networks (PPI Networks)

In this topic, two groups were created: P (protection), aggregating proteins classified as “partial protection” and “protection”, and NP (no protection) for proteins classified as “no protection”. We entered these proteins into STRING v.10 [59] looking for interaction networks associated with them, using *Leishmania* as reference organism. The active interaction sources used were text mining, experiments, database, co-expression, neighborhood, gene fusion and co-occurrence, with medium confidence score (0.400). The networks were built using the target proteins as central nodes and expanding them with the first neighbors, using 500 as maximum value of interactions. From STRING, we obtained the PPI network data and KEGG [60] pathway functional enrichments (*p* < 0.05) of proteins involved in these networks. Then we built the protein networks using Cytoscape [61], adding KEGG information over them.

### 4.6. Statistical Analysis

The analyses were performed using SPSS version 20 (SPSS Inc., Chicago, IL, USA). Association coefficients were determined using Spearman two tailed test and correspondence analysis were determined by Chi-squared, statistical significance was considered when *p* < 0.05.

## 5. Conclusions

In this work, we validated a computational approach regarding epitope prediction, topological structure and pathway analyses to drive a rational vaccine design against leishmaniasis, using antigens tested in murine model described in literature. Our results suggest that CD4^+^ and CD8^+^ T cells are more related to leishmaniasis protection in EO than B cells. For a deeper analysis, we also used PPI networks enriched with KEGG pathways information. According to our results, proteins associated to protection are connected to few pathways when compared to proteins classified as “no protection”. In addition, analysis of PPIs and KEGG pathways associated to proteins from “protection” group corroborate the idea already published in the literature that reverse vaccinology approaches are able to identify proteins related to pathogenicity of infectious agents, helping researchers to understand virulence mechanisms and how immune responses from hosts are able to fight them. Obtained results may be helpful in discovering new potential antigens using computational approaches.

## Figures and Tables

**Table 1:** Selected candidate antigens from dermatotropic and visceratropic *Leishmania* species to leishmaniasis vaccine development. Proteins in literature tested in mice model were selected randomly.

*Leishmania* Tropism	Geographic Area	Specie	Candidate Antigen	Function	MySQL ID	NCBI Sequence Acession	Animal	Experimental Outcome Indicative	Reference
**Dermatotropic *Leishmania* species**	New world	*L. brazilinensis*	Thiol-specific-antioxidant (TSA)	Tryparedoxin peroxidase	LbrM.15.1080	gi 154334618	mice	No protection	[5]
			LeiF	*Leishmania* putative eukaryotic initiation factor	LbrM.25.0580	gi 154338682	mice	No protection	[5]
			LACK	*Leishmania* homolog of receptors for activated C-kinase	LbrM.28.2950	gi 154340729	mice	Partial protection	[5]
		*L. amazonensis*	P4 nuclease partial	Endonuclease activity	A16600	gi 29165287	mice	No protection	[6]
			Cysteine proteinase	Cysteine-type peptidase activity	A22180	gi 30142572	mice	Partial protection	[7]
			HSP20	Heat shock protein	A38570	gi 513044555	mice	No protection	[8]
			GP46	Membrane glycoprotein	A64110	gi 159321	mice	Protection	[9]
		*L. mexicana*	GP63	Metalloendopeptidase activity	LmxM.10.0465	gi 401416782	mice	Protection	[10]
	Old world	*L. major*	LmTSI	Stress-induced protein sti1	LmjF.08.1110	gi 68124434	mice	Protection	[11]
			GP63	Metalloendopeptidase activity	LmjF.10.0470	gi 157865341	mice	Protection	[12]
			PSA 2	Promastigote surface antigen protein 2	LmjF.12.1000	gi 68124979	mice	No protection	[13]
			TSA	Thiol-specific-antioxidant—Tryparedoxin peroxidase	LmjF.15.1080	gi 68125473	mice	No protection	[14]
			Histone H1	DNA binding	LmjF.27.1190	gi 4008565	mice	No protection	[15]
			LACK	*Leishmania* homolog of receptors for activated C-kinase	LmjF.28.2740	gi 157872022	mice	Partial protection	[16]
**Viscerotropic *Leishmania* species**	New world	*L. infantum*	H2A	DNA binding	LinJ.21.1160	gi 339898105	mice	No protection	[17]
			LiCY1	Peptidylprolyl isomerase	LinJ.25.0940	gi 146088699	mice	Partial protection	[18]
			Histone H1	DNA binding	LinJ.27.1070	gi 78146500	mice	No protection	[19]
			CPC	Cysteine-type peptidase activity	LinJ.29.0860	gi 146092987	mice	Protection	[20]
	Old world	*L. donovani*	NH36	Hydrolase activity	LdBPK_181570.1	gi 19697561	mice	Partial protection	[21]
			A2	Amastigote-specific protein—stress response protein	LdBPK_220560.1	gi 12382244	mice	Protection	[22]

**Table 2:** Number of binding Major Histocompatibility Complex (MHC) epitopes, B cell epitopes and subcellular localization predicted by different computational programs.

MySQL ID	Prediction of Binding MHC Epitopes			Prediction of B Cells Epitopes			EO ^1^	Prediction of Subcelular Localization
	Binding MHC Class I Epitopes		Binding MHC Class II Epitopes	AAP12	BCPred12	BepiPred		
	NetMHC	NetCTL	NetMHCII					
LbrM.15.1080	7	74	132	97	32	1	No protection	cyt
LbrM.25.0580	6	31	121	75	23	2	No protection	cyt
LbrM.28.2950	14	67	298	130	15	2	Partial protection	nuc
A16600	4	18	63	21	9	2	No protection	cyt
A22180	15	105	403	214	65	14	Partial protection	ext
A38570	10	46	146	52	0	5	No protection	ext
A64110	28	149	739	193	20	10	Protection	ext
LmxM.10.0465	31	196	747	302	91	36	Protection	ext
LmjF.08.1110	29	177	369	291	79	16	Protection	cyt
LmjF.10.0470	27	177	668	317	52	19	Protection	pla
LmjF.12.1000	23	100	475	226	102	12	No protection	ext
LmjF.15.1080	9	77	199	81	35	5	No protection	cyt
LmjF.27.1190	1	27	89	20	20	2	No protection	nuc
LmjF.28.2740	16	64	301	172	18	7	Partial protection	nuc
LinJ.21.1160	5	52	130	33	30	2	No protection	nuc
LinJ.25.0940	8	26	116	98	69	2	Partial protection	cyt
LinJ.27.1070	1	36	53	80	58	2	No protection	nuc
LinJ.29.0860	21	99	331	201	85	4	Protection	ext
LdBPK_181570.1	14	89	356	161	63	2	Partial protection	ext
LdBPK_220560.1	35	159	669	200	165	12	Protection	pla

^1^ EO = Experimental outcome.

**Table 3:** *Leishmania* predicted proteomes used in the study. The version and number of predicted proteins of each species are shown.

*Leishmania* Specie	Version of Proteome	Predicted Proteins
*L. braziliensis*	3.1	8357
*L. amazonensis*	- ^1^	8168
*L. mexicana*	9.0	8250
*L. major*	9.0	8400
*L. donovani*	8.0	8083
*L. infantum*	3.2	8241

^1^ This is a draft version. This proteome still has many annotation errors.

## Supplementary Materials

Supplementary materials can be found at www.mdpi.com/1422-0067/18/2/371/s1.
