# Supplementary material for: Immunoinformatics Features Linked to Leishmania Vaccine Development: Data Integration of Experimental and In Silico Studies
Source: Int J Mol Sci. 2017 Feb 10;18(2):371. doi: 10.3390/ijms18020371 (PMC5343906; doi:10.3390/ijms18020371)
Supplement: Supplementary file 1 [file ijms-18-00371-s001.pdf]

Supplementary Materials: Immunoinformatics Features Linked to *Leishmania* Vaccine Development: Data Integration of Experimental and In Silico Studies

Rory C. F. Brito, Frederico G. Guimarães, João P. L. Velloso, Rodrigo Corrêa-Oliveira, Jeronimo C. Ruiz, Alexandre B. Reis and Daniela M. Resende

Table S1. Statistical Package for the Social Sciences (SPSS) output showing values of inertia and significance (Sig.) for experimental outcome and number of predicted epitopes for T CD4$^+$ cells.

Dimension	Singular Value	Inertia	Chi Square	Sig.	Proportion of Inertia		Confidence Singular Value	
					Accounted for	Cumulative	Standard Deviation	Correlation 2
1	0.672	0.452			0.643	0.643	0.143	0.003
2	0.501	0.251			0.357	1.000	0.181	
Total		0.703	14.756	0.022 [a]	1.000	1.000		

[a] Six degrees of freedom.

Table S2. SPSS output showing values of inertia and significance (Sig.) for experimental outcome and number of predicted epitopes for CD8$^+$ T cells.

Dimension	Singular Value	Inertia	Chi Square	Sig.	Proportion of Inertia		Confidence Singular Value	
					Accounted for	Cumulative	Standard Deviation	Correlation 2
1	0.808	0.653			0.918	0.918	0.105	0.087
2	0.242	0.059			0.082	1.000	0.230	
Total		0.711	14.933	0.021 [a]	1.000	1.000		

[a] Six degrees of freedom.

Table S3. SPSS output showing values of inertia and significance (Sig.) for experimental outcome and number of predicted epitopes for B cells.

Dimension	Singular Value	Inertia	Chi Square	Sig.	Proportion of Inertia		Confidence Singular Value	
					Accounted for	Cumulative	Standard Deviation	Correlation 2
1	0.632	0.400			0.753	0.753	0.136	−0.078
2	0.362	0.131			0.247	1.000	0.175	
Total		0.531	11.148	0.084 [a]	1.000	1.000		

[a] Six degrees of freedom.
